# Investigation and Statistical Modeling of the Mechanical Properties of Additively Manufactured Lattices

**DOI:** 10.3390/ma14143962

**Published:** 2021-07-15

**Authors:** Derek G. Spear, Anthony N. Palazotto

**Affiliations:** Air Force Institute of Technology, Wright-Patterson AFB, Dayton, OH 45433, USA; anthony.palazotto@afit.edu

**Keywords:** mechanical properties, additive manufacturing, Triply Periodic Minimal Surfaces (TPMSs), lattices, material design characteristics, Design of Experiments (DOE)

## Abstract

This paper describes the background, test methodology, and experimental results associated with the testing and analysis of quasi-static compression testing of additively manufactured open-cell lattice structures. The study aims to examine the effect of lattice topology, cell size, cell density, and surface thickness on the mechanical properties of lattice structures. Three lattice designs were chosen, the Diamond, I-WP, and Primitive Triply Periodic Minimal Surfaces (TPMSs). Uniaxial compression tests were conducted for every combination of the three lattice designs, three cell sizes, three cell densities, and three surface thicknesses. In order to perform an efficient experiment and gain the most information possible, a four-factor statistical experimental design was planned and followed throughout testing. A full four-factor statistical model was produced, along with a reduced interactions model, separating the model by the significance of each factor and interaction terms. The impact of each factor was analyzed and interpreted from the resulting data, and then conclusions were made about the effects of the design parameters on the resultant mechanical performance.

## 1. Introduction

Open-cell periodic cellular structures, or lattices, continue to gain interest as an engineering material as a method for light-weighting structures or energy absorbing and control applications. These novel engineered cellular materials, where lattice structures are considered, are a fledgling category of materials [1]. However, modern advancements in manufacturing methods, namely additive manufacturing, have made the use of lattice structures more feasible.

In order to determine the utility of lattice designs, their mechanical properties must be able to be readily determined. Prior research on cellular structures found that three primary factors influence the mechanical response of these materials: the material properties of the base material from which the lattice is fabricated, the relative density of the structure, and the lattice design or topology [2]. Thus, when designing periodic cellular structures, a handful of variables can be directly controlled: the base material, topology, relative density, cell size, cell density, and cellular surface thickness. Presented here is a brief introduction to the factors under analysis, with lattice design covered in detail in the Methodology section.

**Relative Density** ($\rho_{rel}$). The ratio of the density of the cellular structure ($\rho^{*}$) relative to the base, or fabrication, material density ($\rho_{s}$), see Equation (1). Relative density is set at the unit cell level and is contingent upon the cell topology, cell size, and surface thickness. Changes made to any of these parameters will affect the lattice’s relative density. For example, if the topology and cell size are maintained constant, but the surface thickness is increased, the relative density of the lattice will also increase since there is more material present within the cell. Likewise, if the topology and surface thickness are maintained constant, but the cell size increases, the relative density will decrease as there is less material present within the cell bounds.
  (1) $$\rho_{rel}=\frac{\rho^{*}}{\rho_{s}}$$
**Cell Size.** The measured cell cross-sectional distance or height.
**Cell Density.** The number of cells replicated through the structure cross-sectional distance or height.
**Surface Thickness.** The mean thickness of the lattice cell structure; the through-surface thickness of a surface-based lattice or cross-sectional dimension of a strut-based lattice.

This research is focused on lattice structures, which are open-cell periodic cellular structures. Here, open-cell refers to structures characterized by an interconnected network of open space, and periodic refers to structures that are fashioned through the replication of a unit cell design. Furthermore, the lattice classification can be split into strut-based lattice designs and surface-based lattice designs [3]. Strut-based designs are identified by a joint and frame architecture of its structural members that deforms through stretching to carry a load, similar to a truss. Surface-based designs are identified by a sheet-like architecture, which deforms through bending, buckling, or crushing, similar to foams.

The differences in mechanical behavior and failure modes suggest that each network type would perform better within different applications. The loading and failure methods of strut-based networks indicate that these lattice types perform better under uniaxial loading and present with a higher strength-to-weight ratio [2,4]. The loading and failure of surface-based networks produce a more significant plateau response, the region between yield and densification, which indicates that the surfaced-based network would perform better within energy absorbing applications [2]. With a primary area of interest being the energy absorbing capabilities of metal lattices, the primary focus here will be further restricted to surface-based lattice designs.

**Figure 1:**
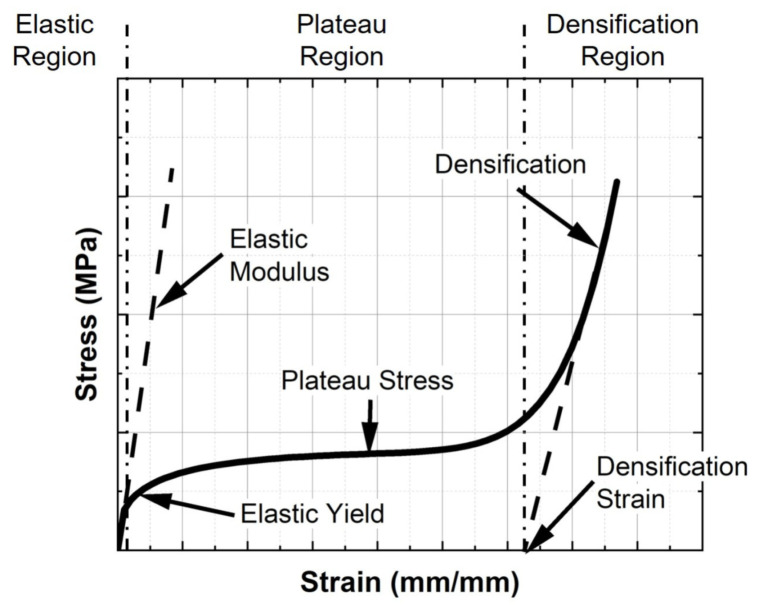
Typical engineering stress-strain response of a surface-based lattice structure.

Figure 1 depicts the characteristic engineering stress-strain response curve of a surface-based lattice obtained through uniaxial compression testing. There are three distinct response phases present within this curve. First, under initial loading, the response displays a linear-elastic relationship up to its elastic yield strength. Here the Elastic Modulus (*E*) of the lattice design can be determined, along with the Yield Strength ($\sigma_{y}$). The second phase of the response represents the plastic response of the lattice, where cell failure and collapse occur, and here the plateau stress ($\sigma_{pl}$) is determined. The final phase present in the response curve is regarded as the densification region, which is characterized by a sharp rise in stress due to the reduction of void space within the lattice. In this region, the material responds in a manner similar to a porous solid.

Lattice architectures have been tested across various materials [3,5,6,7,8]; however, there has not been significant research into characterizing the mechanical properties of additively manufactured Inconel 718 (IN718) lattices. Wadley et al. performed some of the initial mechanical characterizations of lattices, exploring strut-based and surface-based lattices manufactured utilizing traditional means [9]. Murr et al. evaluated the additive manufacturing processes for metals and alloys, including IN718, assessing the microstructural effects on the material’s mechanical properties [10]. Körner expanded this research through the exploration and characterization of additive manufacturing methods and materials through the fabrication of thin-walled surfaces, strut-based lattices, and other cellular designs [11]. Huynh et al. extended the evaluation of microstructure effects to additively manufactured IN718 micro-trusses, comparing the precipitate structure to that of wrought IN718 [12]. Al-Ketan et al. performed testing across various strut, skeletal, and surfaced-based lattices, additively manufactured out of steel variants, focusing on determining the change in mechanical properties across cell design types [3,7,13]. Recently, research has expanded into the evaluation of additively manufactured IN718 surface-based lattices, to include novel variations on the base lattice topology [14,15,16]. These works focused primarily on macro-scale evaluation of the energy absorbing characteristics of the lattice cell designs. Bodaghi et al. detailed a closer examination of the energy absorption aspect of additively manufactured polymers, using tunable dual-media sandwich lattice structures in reversible energy absorption applications [17,18]. Their research tuned material response through manipulation of the fabrication materials and their shape memory properties. The current research effort provides a more in-depth examination of four lattice design variables (topology, cell size, cell density, and cellular surface thickness) through the use of statistical analysis. This analysis aims to provide further insight into the effects of each parameter, as well as their interactions, on the mechanical properties of several additively manufactured IN718 lattices.

## 2. Methodology

### 2.1. Experimental Setup and Procedures

#### 2.1.1. Lattice Designs

Three lattice cell designs were evaluated as part of this study, all of which were based on Triply Periodic Minimal Surfaces (TPMS). TPMS are unique structures that meet three distinct criteria: being periodic in all three axes, having minimal surface area within the bounded region, and having zero mean curvature [19]. Mathematically, mean curvature can be defined as the average principal curvature of the surface [20].

The TPMS designs chosen were the Diamond (Schwarz D) surface, the Schoen I-WP surface, and Primitive (Schwarz P) surface. The unique nature of TPMS lattices is that with their architecture being periodic along all three dimensions, the base structure can be easily replicated to fill an engineering design space. Furthermore, each of these lattice designs can be represented by trigonometric functions, presented in Equations (2)–(4) for the Diamond, I-WP, and Primitive lattices, respectively [21]. The ability of these designs to be modeled through analytic equations increase their utility in computer aided design (CAD) and fabrication through additive means. In the TPMS equations, *x*, *y*, and *z* are the Cartesian coordinates in three-dimensional space, and *m* serves as a periodicity scaling factor, calculated as the ratio of the desired cell size over π. This scaling factor sets the cell density within the prescribed design space within the trigonometric representation of the lattice.

(2) $$\begin{array}{cc} \; & \sin\left(mx\right)\sin\left(my\right)\sin\left(mz\right)+\sin\left(mx\right)\cos\left(my\right)\cos\left(mz\right)\\ \; & +\cos\left(mx\right)\sin\left(my\right)\cos\left(mz\right)+\cos\left(mx\right)\cos\left(my\right)\sin\left(mz\right)=0 \end{array}$$

(3) $$\begin{array}{cc} \; & 2\left[\cos\left(mx\right)\cos\left(my\right)+\cos\left(mx\right)\cos\left(mz\right)+\cos\left(my\right)\cos\left(mz\right)\right]\\ \; & -\cos\left(2mx\right)\cos\left(2my\right)\cos\left(2mz\right)=0 \end{array}$$

(4) cos(mx)+cos(my)+cos(mz)=0

**Figure 2:**
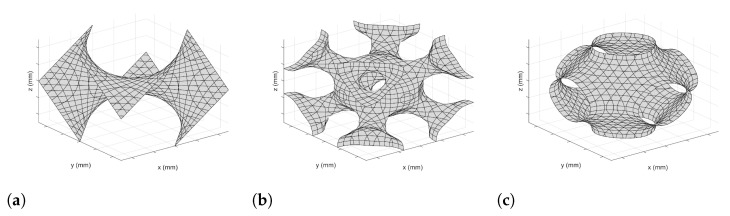
Thin-surfaced TPMS lattice designs: (**a**) Diamond cell, (**b**) I-WP Cell, (**c**) Primitive cell.

A depiction of each of the three lattice cells is presented in Figure 2.

#### 2.1.2. Additive Manufacturing

All of the specimens used in this study were fabricated by selective laser melting (SLM) of gas atomized spherical IN718 powder. Table 1 presents the chemical composition of the IN718 powder.

Fabrication was completed using a General Electric Concept Laser M2 SLM machine equipped with a 400 W continuous-wave ytterbium fiber laser. The SLM fabrication process spread a thin layer of powder across the build surface where the laser melted and fused the powder according to the determined scan strategy. This process was repeated layer by layer until the build was completed. Within the manufacturing process, six parameters could be controlled to set the build environment: laser power, scan speed, laser spot size, hatch or scan spacing, contour offset, and powder layer thickness. Varying these parameters would lead to significant changes in the part microstructure, affecting its resultant material properties. Build strategies are generally based upon the physical location within the designed part. For example, the surface sections of the part will typically use a lower laser power setting to achieve a lower surface roughness. In contrast, the interior areas of the part will use a higher laser power and scan speed to increase productivity. Since these lattices were thin surfaces, they were manufactured using the surface finish parameters: 120 W laser power, 280 mm/s scan speed, 50 µm spot size, and a 90 µm contour offset. Contour offset defined the distance from the CAD model’s external surface to the midpoint of the laser scan path and was based on the material melt pool width.

#### 2.1.3. Mechanical Testing

**Figure 3:**
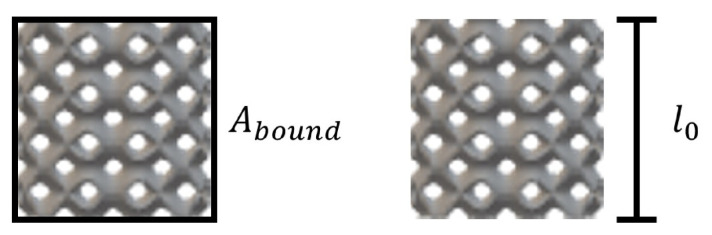
Depiction of the bounding cross-sectional area and original height of a lattice specimen.

Before undergoing mechanical testing, the specimen dimensional measurements and mass were recorded to determine the relative density of the specimen and verify the fabrication parameters against the desired specimen parameters. The specimen’s density was calculated as the mass divided by the specimen bounding volume. The bounding volume was calculated as the bounding cross-sectional area multiplied by the original specimen height, these reference dimensions are presented in Figure 3. The specimen’s relative density was then determined through Equation (1). Within each lattice cell design, the relative density was primarily determined by the relationship between the cell size and surface thickness.

Mechanical testing was accomplished following the well-established practices specified by the American Society for Testing and Materials (ASTM) E9-19 for quasi-static uniaxial compression testing of metals. One exception was made to these procedures, in that cubes with a one-to-one length-to-diameter ratio were used instead of the prescribed cylinders [22]. Cubes were used based on the previous testing of cylindrical lattice specimens, where all of the specimens presented a buckling response versus uniaxial compression [14,15]. In general, the uniaxial compression testing process consisted of loading the specimen under an increasing axial compression force while recording both force and displacement data to determine the stress-strain response of the material. In this study, displacement control was utilized to achieve a target strain rate within the quasi-static regime. All of the testing for this research was performed on an MTS Systems Corporation Model 810 servo-hydraulic universal testing machine. The precision of the MTS machine as configured during testing was 0.001 mm in displacement and 13 N in force.

Once the force and displacement data were obtained, the specimen’s engineering stress-strain response was calculated. First, the recorded force was transformed into engineering stress by dividing the applied load by the bounding cross-sectional area of the specimen. In the case of the cube specimens, that was the overall cube width squared. Next, the engineering strain was calculated as the change in specimen length, or recorded displacement, divided by the original specimen length. Once the stress-strain response for the specimen was determined, it was used to calculate the mechanical properties of each design.

Four mechanical properties of interest were determined for each lattice: the modulus of elasticity, yield strength, plateau stress, and toughness. The modulus of elasticity describes the elastic deformation of the lattice up to the proportional limit where yield occurs, and was found as the slope of the linear elastic region of the engineering stress-strain response. The yield strength is the point at which plastic deformation begins within the lattice, and is dependent on the deformation pattern of the lattice. While, due the nature of being thin-walled structures, all three lattice designs are subject to through-surface cracking, the Diamond and Primitive cells tend to undergo shear deformation, while the I-WP is more subject to successive layer collapse [16]. The yield strength was determined using the 0.02% Offset method. The plateau stress also signifies the deformation modes of the lattice, and was obtained by finding the mean stress values between 20% and 40% strain. Within the plateau response, bending dominated lattices tend to exhibit a near constant stress values, where stretch dominated lattices generally display a more oscillatory response. Finally, the toughness was calculated as the area beneath the stress-strain response from load initiation up to the densification strain. Toughness was chosen as a metric of interest due to its relationship with the energy absorbing capability of the structure.

### 2.2. Statistical Experimental Design

Previous experimentation was used to identify the defining parameters that govern the mechanical response of the lattices under compression [14,15,16]. A Design of Experiments (DOE) study will be conducted to characterize these variables by identifying and quantifying which defining parameters are significant. The DOE study will quantify the significance of each parameter individually, and identify the significance of interactions between the variables on the model responses as a whole. A series of experiments must be conducted to obtain the data necessary to be analyzed using the statistical method known as analysis of variance (ANOVA). ANOVA is a method used to determine the differences between multiple sets of data averages by comparing the mean responses to factor manipulations within a controlled experimental environment. This method also determines the impact of the individual factors through analysis of the means of different applied factors, or treatments.

In most cases, a factorial design is conducted to determine the experiments that must be conducted to attain sufficient data to make justifiable claims on the significance of the defining parameters, also referred to as factors. When a DOE study is planned with a substantial number of factors, generally considered greater than three factors, a standard factorial design chosen is an unreplicated $l^{k}$ design. This design takes each factor, *k*, evaluated across *l* levels to quantify the effect of the factor on the output responses. After the requisite data is collected, the ANOVA is performed on the factors and their interactions to determine factor significance and develop a regression fit. Finally, the regression fit is determined using the method of least squares to create a linear equation to fit the model data acquired from the experiments performed during the factorial analysis [23].

The statistical design used for this experiment was a three-level full factorial design, $3^{k}$, in which all possible combinations for the *k* factors are represented within the experiment and randomly decided for each experimental treatment. For this research effort, experimental factors considered were the lattice design, cell size, cell density, and surface thickness. The factor levels for the lattice design were the Diamond, I-WP, and Primitive lattice. The three levels used for the cell size were 4 mm, 6 mm, and 8 mm. In the case of the cell density, the factor levels were 3, 4, and 6 unit cells across the width of the specimen. Finally, for the surface thickness, the three levels were 200 µm, 400 µm, and 600 µm. Each level was then numerically coded as 0, 1, and 2 for the low, intermediate, and high factor levels. The actual and coded factor levels are presented in Table 2. When considering this design, with l=3 levels for each of the k=4 factors, the experimental design results in $3^{4}=3\times 3\times 3\times 3=81$ required experimental treatments for a single data set.

Only a single replication of data was taken, executed as a Completely Randomized Design (CRD), where the selection of applied factors from within the available choices is applied at random for the given experiment. The test matrix with run order was generated utilizing a random number generator for each possible combination of the parameters then sorted by ascending order. The experiment was conducted following the developed test matrix using a strain rate of 0.002 s^−1^, recording data at a rate of 100 Hz, then post-processing the data to ensure it was taken within quasi-static tolerances.

A general effects based model was developed for this CRD design, including the four factors described above. This initial model assumes all potential terms are significant, and therefore also includes the potential interaction terms. The initially proposed experimental model is shown in Equation (5) with a treatment structure expressed in the over parameterized form:

(5) $$\begin{array}{cc} \; & y_{ijkl}=\alpha_{i}+\beta_{j}+\gamma_{k}+\zeta_{l}+{\left(\alpha \beta \right)}_{ij}+{\left(\alpha \gamma \right)}_{ik}+{\left(\alpha \zeta \right)}_{il}+{\left(\beta \gamma \right)}_{jk}\\ \; & +{\left(\beta \zeta \right)}_{jl}+{\left(\gamma \zeta \right)}_{kl}+{\left(\alpha \beta \gamma \right)}_{ijk}+{\left(\alpha \beta \zeta \right)}_{ijl}+{\left(\beta \gamma \zeta \right)}_{jkl}+\varepsilon_{ijkl} \end{array}$$

In this model, $y_{itjk}$ represents the data point resulting from the treatment defined by the combination of the *i*th level of factor one, the *j*th level of factor two, the *k*th level of factor three, and the *l*th level of factor four. $\alpha_{i}$ represents factor one (lattice design) with i=3 levels, $\beta_{j}$ represents factor two (cell size) with j=3 levels, $\gamma_{k}$ represents factor three (Cell Density) with j=3 levels, and $\zeta_{l}$ represents factor four (surface thickness) with l=3 levels. (αβ), (αγ), (αζ), (βγ), (βζ), (γζ), (αβγ), (αβζ), and (βγζ) represent each of the possible interaction terms, and the remaining term in the model, $\varepsilon_{ijkl}$, is the random effects elements, representing variation within the experiment. The initial experimental model can also be written in matrix notation and expressed in the full rank parameterized form for the complete experiment in Equation (6).

(6) $$\begin{array}{cc} \; & y=X_{\alpha }\alpha +X_{\beta }\beta +X_{\gamma }\gamma +X_{\zeta }\zeta +X_{\left(\alpha \beta \right)}\left(\alpha \beta \right)+X_{\left(\alpha \gamma \right)}\left(\alpha \gamma \right)+X_{\left(\alpha \zeta \right)}\left(\alpha \zeta \right)+X_{\left(\beta \gamma \right)}\left(\beta \gamma \right)\\ \; & +X_{\left(\beta \zeta \right)}\left(\beta \zeta \right)+X_{\left(\gamma \zeta \right)}\left(\gamma \zeta \right)+X_{\left(\alpha \beta \gamma \right)}\left(\alpha \beta \gamma \right)+X_{\left(\alpha \beta \zeta \right)}\left(\alpha \beta \zeta \right)+X_{\left(\beta \gamma \zeta \right)}\left(\beta \gamma \zeta \right)+\varepsilon \end{array}$$

## 3. Results and Discussion

### 3.1. Experimental Results

**Figure 4:**
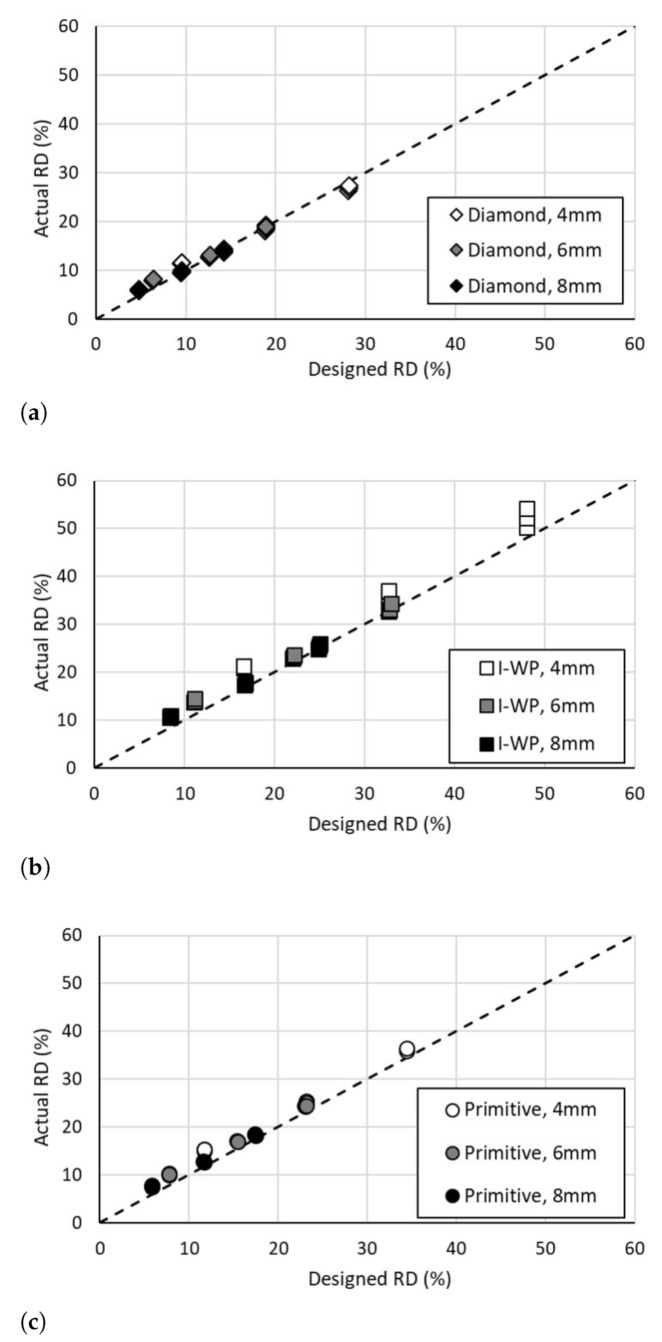
Designed versus actual relative density of: (**a**) Diamond lattice design, (**b**) I-WP lattice design, (**c**) Primitive lattice design.

As mentioned, before compression testing, each of the fabricated specimens was measured and weighed to determine their actual relative densities. A separate comparison plot was developed for an individual lattice design with the actual, as fabricated, relative density plotted against the designed relative density from the CAD model, Figure 4. Within Figure 4, with the Diamond design variations depicted in Figure 4a, the I-WP design variations in Figure 4b, and the Primitive design variations in Figure 4c. The relative density range for the I-WP lattice was higher than the ranges for the Diamond and Primitive lattices; this is primarily due to the cell topology. As an example of this difference, with a cell size of 4 mm, cell density of 3, and surface thickness of 400 µm, the Diamond lattice’s relative density was 18.34%, the I-WP’s relative density was 32.64%, and the Primitive lattice had a relative density of 24.31%. The difference between the actual and designed relative density can be attributed to several factors: intricacy of the design, variation in fabricated surface thickness, and bonding of loose powder to the melt surface. Some of the fabrication parameters, such as laser beam size and powder, are leading causes for variation in surface thickness. These laser parameters set the size of the melt pool, which in turn sets the accuracy of the scan pattern and precision of surface features. The size of the melt pool also affects the amount of powder that inadvertently bonds to the specimen.

**Figure 5:**
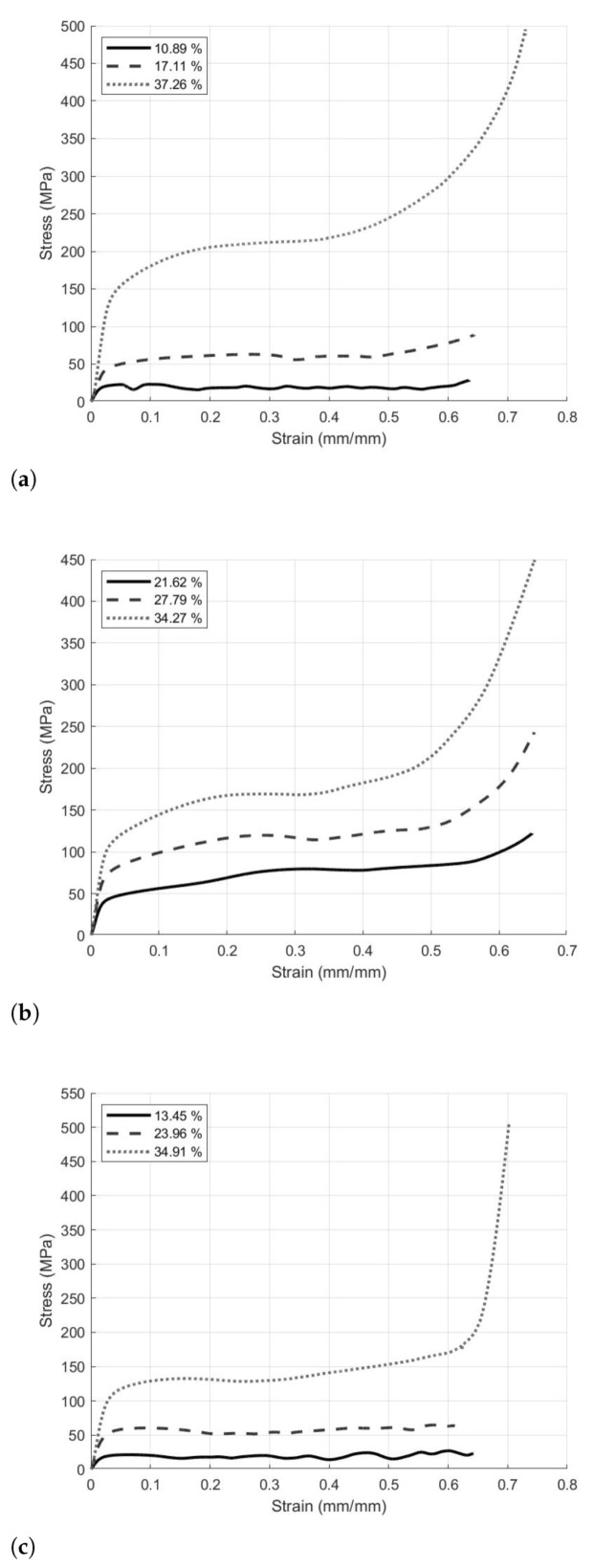
Representative stress-strain response curves of: (**a**) Diamond lattice design, (**b**) I-WP Lattice design, (**c**) Primitive lattice design.

Once the force and displacement data were converted to engineering stress and strain, the response curves were plotted, and the compressive mechanical properties of the lattices were determined. Each of the calculated properties was plotted against the relative density to compare results across lattice designs. Representative stress-strain response curves are presented in Figure 5. For each lattice design, there was a discernible change in the elastic modulus with change in relative density, in that a higher relative density produces a higher elastic modulus. The same relationship could easily be seen for the yield strength and plateau stress, and as a result of the higher plateau stress there was also a higher toughness value. The plateau regions of the I-WP responses, Figure 5b, displayed minimal oscillations indicating a bending dominated response over this relative density range. However, for the Diamond and Primitive lattices, Figure 5a,c, the lower relative density responses displayed more prevalent oscillations, indicative of stretch dominated deformation, while the upper relative density response displayed a relatively smooth plateau response, again indicating bending dominated deformation [4,16]. This signifies that the deformation response within the plateau region could vary with relative density.

**Figure 6:**
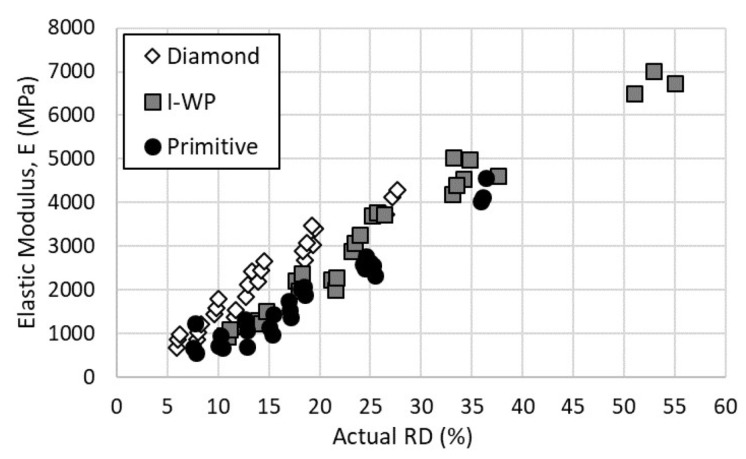
Modulus of elasticity versus actual relative density of lattice designs.

The modulus of elasticity is presented in Figure 6. All three of the lattices displayed a linear relationship between the elastic modulus and relative density. Across the shared relative density range, 10–20%, the Diamond lattice exhibited the highest modulus of elasticity, followed by the I-WP and Primitive lattices closely aligned together. As the relative density increased, the I-WP elastic modulus separated from that of the Primitive design.

**Figure 7:**
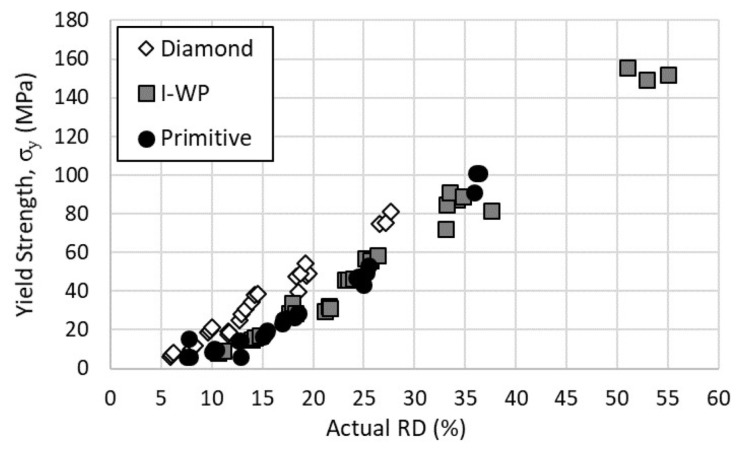
Yield strength versus actual relative density of lattice designs.

Figure 7 displays the results for the yield strength across the tested specimens. The Diamond and I-WP lattices exhibited a linear relationship between the yield strength and relative density, as with the elastic modulus; however, the Primitive lattice appeared to display a quadratic relationship below 20% relative density. This change in relationship was likely due to a change in deformation behavior with the increase in relative density. Again, the Diamond lattice provided the highest yield strength over the shared relative density range, where the I-WP and Primitive lattice designs provided nearly identical results.

**Figure 8:**
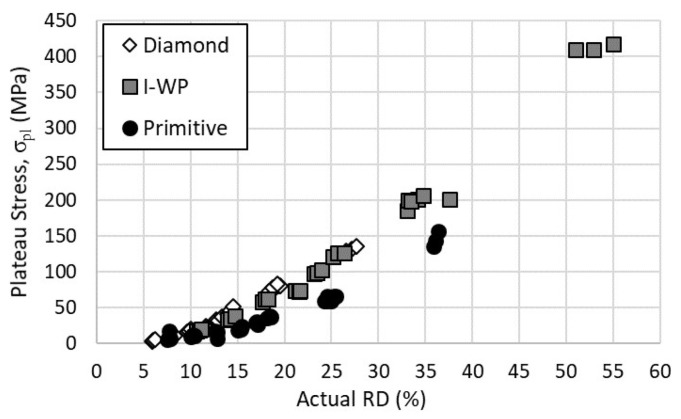
Plateau stress versus actual relative density of Lattice Designs.

The results for the plateau stress are shown in Figure 8. All three lattice designs appeared to feature a quadratic relationship between the determined plateau stress and relative density. In contrast to the elastic modulus and yield strength, the I-WP lattice demonstrated plateau stress values near that of the Diamond designs. However, the Primitive lattice still provided the lowest yield strength of the three designs.

**Figure 9:**
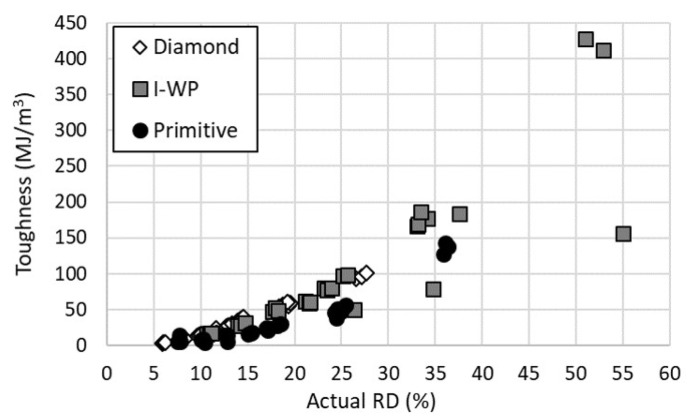
Toughness versus actual relative density of lattice designs.

The results for the toughness of the specimens, Figure 9, were similar to that of the plateau stress. This result was not unanticipated, as the plateau stress is the stress value across the majority of the response region integrated to find the toughness. Again, all three lattices appeared to display a quadratic relationship between the toughness and actual relative density. The Diamond and I-WP provided nearly identical results, except for two outliers in the I-WP results. These two outliers were due to a lower densification strain within the two experiments. As with the modulus of elasticity, with increasing relative density, there was a separation in performance between the I-WP and Primitive lattices.

### 3.2. Four-Factor ANOVA Decomposition

An ANOVA decomposition was accomplished for this full factorial design. Table 3 displays the ANOVA structure with equations shown for degrees of freedom, sums of squares, and mean squares. Within the ANOVA tables, Factor A refers to the lattice design, Factor B is the cell size, Factor C the cell density, and Factor D is used for the surface thickness.

A MATLAB script was generated to perform the calculations for degrees of freedom, sums of squares, and mean squares presented in the ANOVA structure table. The resultant ANOVA values are provided in Table 4, Table 5, Table 6 and Table 7, which show the entire plot of factors and interaction terms. Additionally, F tests for significance were accomplished for each factor, including the interaction terms. F-statistic values, and the associated P values, are also shown in the table. Finally, a conclusion column was added to the ANOVA table to indicate the significance of the corresponding item in the table. These conclusions enabled the creation of a reduced model using only significant factors, which provided more degrees of freedom for the residuals.

Based on the ANOVA decomposition, it was determined that the three-source interaction terms, and the two-source interaction terms that included the cell density, Factor C, were not significant across all of the material properties and were therefore removed from the final model. The final reduced model, Equation (7), only included terms initially determined to be statistically significant. As a result, the associated final ANOVA results are shown in Table 8, Table 9, Table 10 and Table 11 for the elastic modulus, yield strength, plateau stress, and toughness.

(7) $$\begin{array}{ccc} \; & y=X_{\alpha }\alpha +X_{\beta }\beta +X_{\gamma }\gamma +X_{\zeta }\zeta +X_{\left(\alpha \beta \right)}\left(\alpha \beta \right)+X_{\left(\alpha \zeta \right)}\left(\alpha \zeta \right)+\; & +X_{\left(\beta \zeta \right)}\left(\beta \zeta \right)+\varepsilon \end{array}$$

The least squares estimates of effects were determined using the reduced model, Equation (7), and the contrasts from this analysis are presented in Table 12. As these estimates were contrasts, the values within the table represent the difference from the overall response mean. The mean response values are also presented in Table 12. The mean error of the model, considering all four of the material properties, was −9.53 × 10${}^{-15}$. Additionally, confidence intervals were computed for the mechanical property estimates using the reduced interaction model, shown in Table 13. The confidence intervals were based on a t-distribution and were determined utilizing the residual degrees of freedom and mean square value.

**Figure 10:**
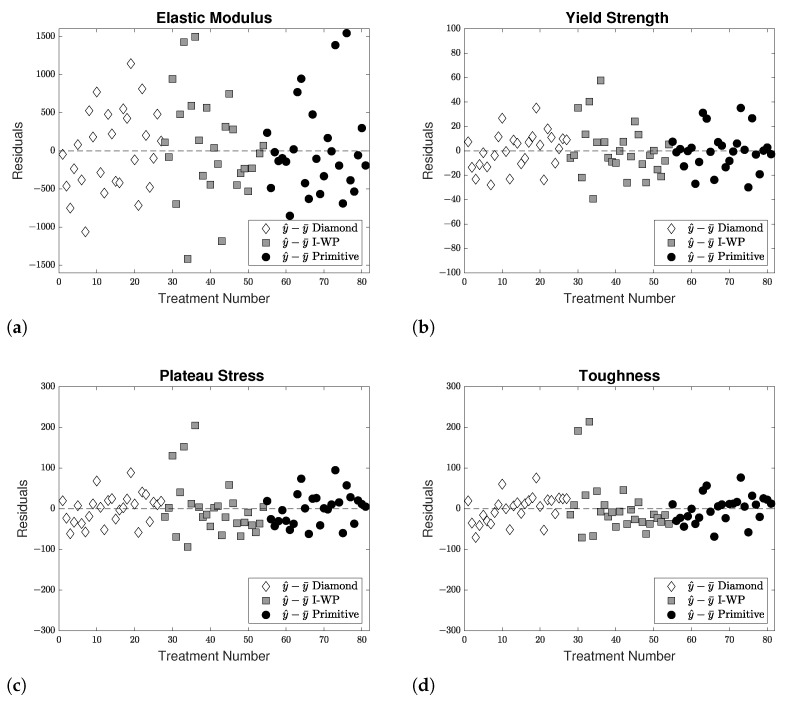
Residuals plotted against treatment number for: (**a**) elastic modulus, (**b**) yield strength, (**c**) plateau stress, (**d**) toughness.

Furthermore, the residuals were analyzed and plotted in Figure 10 against the treatment number. In the residuals plots for the plateau stress and toughness, Figure 10c,d, a few data points appeared to have significant residuals values; however, these points correlated to the I-WP specimens with relative densities greater than 50%. At relative densities that high, the material no longer behaved as a lattice, but rather a porous solid [24]. Thus, there appeared to be no unusual findings concerning the residuals, validating independence and constant variance assumptions. For reference, the residual ranges depicted in these figures correlated to a maximum error of 10–15%, depending on the property under evaluation.

**Figure 11:**
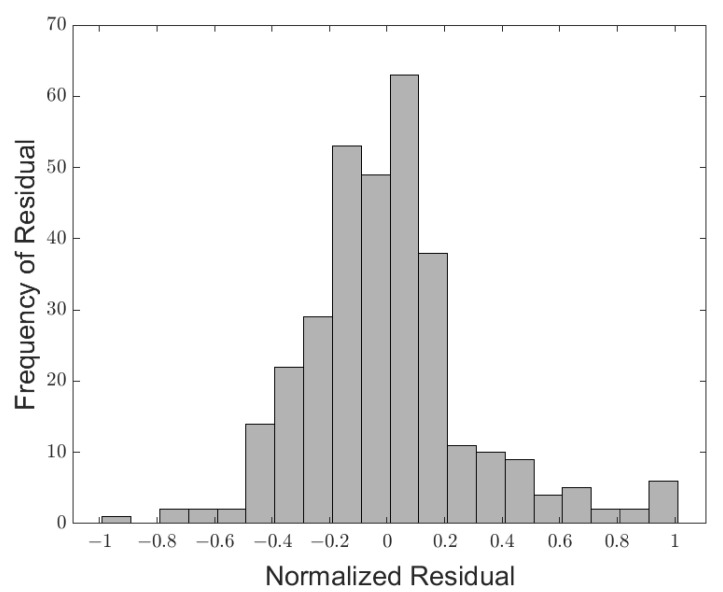
Histogram of normalized residuals.

Finally, the data were analyzed to assure normality by plotting a histogram of the residuals. The assumption of normal error was satisfied if the histogram portrayed a normal distribution centered around zero. As seen in the resultant histogram, Figure 11, the distribution of residuals was approximately normal and centered about zero, but the right tail was slightly thicker than the left tail. However, when using a fixed effects model for ANOVA, moderate departures from normality are not a significant cause for concern as the F-tests are only slightly affected [25]. Therefore, both the analysis and contrasts were considered robust to the normality assumption.

### 3.3. Interpretation of Results

The results show that three of the main effects—lattice design, cell size, and surface thickness—are significant at the α=0.01 level for all four material properties investigated. However, the p-values within the reduced model indicate that cell density is only significant to the modulus of elasticity for the lattice.

A review of the estimates and contrasts, Table 12, reveals several insights. In evaluating the effects of lattice design across all four of the mechanical properties determined, the I-WP design provides the highest property values. This is likely due to the link between the cell’s topology to its relative density, and the relationship between the lattice’s relative density and material properties, as shown in Figure 6, Figure 7, Figure 8 and Figure 9. The Diamond design estimates show that it provided a higher elastic modulus than the Primitive design; however, for the other three properties, the Diamond and Primitive designs provided similar results. Concerning the cell size factor, a smaller cell size yielded better performance for all four properties, which is likely due to the lattice at a given relative density supporting a similar load over a smaller individual cell area. As mentioned, if all other factors remain constant and the cell size decreases, the relative density will increase, and the resultant mechanical properties will reflect this change. Cell density was the least significant of the main factors, displaying the smallest factor effect estimate range for each property. However, the cell density factor did demonstrate consistent influence for the mechanical properties, where a higher density value generated superior mechanical performance. By spreading the load across an increased number of cells, the lattice deformation mechanisms are likely delayed, which generates the increase in mechanical performance. The final factor, surface thickness, had the highest impact on mechanical performance; this is evidenced through the most extensive range of effects estimates for the properties. Considered as a single factor change, increased surface thickness leads to an increased relative density, which produces higher mechanical property values.

Beyond the primary factors of interest, all of the interactions highlighted in Table 8, Table 9, Table 10 and Table 11 proved significant in the achieved results. All three of the factors present within the interaction terms can be tied to the relative density of the structure [26]. Due to the significant difference in the topology of the lattice designs, it is not surprising that the interaction factors that dealt with the design were significant in determining the mechanical properties. Furthermore, the relationship between the surface thickness and cell size has been used to characterize the relative density when evaluated by individual lattice topology [3,7,13].

Further inspection of the reduced model ANOVA tables, in particular the F-test results, provides further understanding of the individual importance of each factor with regard to the mechanical properties. For the elastic modulus, Table 8, the F-test values show that the surface thickness, Factor D, has the most significant influence on the modulus value. The difference in F-test value for the cell size and lattice design factors indicates that changing these factors provides similar resultant changes in the modulus. The interaction factors for the lattice design with surface thickness and cell size with surface thickness have a more significant impact on the lattice elastic modulus than cell density. The results for the yield strength, Table 9, provide similar insights for the main factors; however, the difference between the F-test value for cell size and lattice design is more prominent, with the cell size value indicating more significant influence over the material property. The trends noticed for plateau stress and toughness, Table 10 and Table 11, are nearly identical, with the surface thickness still being the most significant factor. However, in the case of these two properties, the lattice design has a slightly higher F-test value than the cell size, which indicates that topology plays a more significant factor in the plateau stress and densification than the cell size. Additionally, the spread in F-test values between the lattice design, cell size, and surface thickness is much lower than the elastic modulus and yield strength, indicating a more even distribution of factor effects. This finding is significant, as it suggests that several possible combinations of these factors could be used to achieve a target property value. This discovery opens the design trade space to secondary effects outside of the primary application goal considerations.

## 4. Conclusions

This experimental campaign and statistical analysis investigating the influence of lattice topology, cell size, cell density, and surface thickness has revealed statistically significant effects attributable to all four factors when considering the mechanical response of the specimens under uniaxial compression. Furthermore, least squares estimates of effects indicate that the lattice surface thickness provides the most significant impact and cell density of the specimens provides the most negligible impact on the subsequent material properties.

However, this is a unique analysis effort, where evaluation between the contrasts of these four design parameters has been made possible. Three statistically significant interactions between main effects were observed, all of which can be linked as factors in the relative density of the specimen. While this is not an entirely new finding, neither is it a trivial finding, since it speaks volumes to the underlying connection between these factors in determining the mechanical response of the lattice structure. Furthermore, the observation of multiple factor combinations achieving the same material property results opens up the trade space between these factors within the design stage. This finding will allow for primary and secondary effects to be considered when designing a lattice structure, especially in energy absorbing applications.

## Figures and Tables

**Table 1:** Chemical composition of the IN718 powder.

Element	Ni	Cr	Cb + Ta	Mo	Ti	Al	Co	Mn	Si	Cu	C	P	S	B	Fe
% Weight (Min-Max)	50–55	20.5–23	4.75–5.50	2.8–3.3	0.65–1.15	0.2–0.8	0–1	0–0.35	0–0.35	0–0.3	0–0.08	0–0.015	0–0.015	0–0.006	Balance

**Table 2:** Experimental design levels in actual and coded units.

Parameter	Units	3 Levels		
		0	1	2
Lattice Design	-	Diamond	I-WP	Primitive
Cell Size	mm	4	6	8
Cell Density	-	3	4	6
Surface Thickness	µm	200	400	600

**Table 3:** ANOVA structure and equations for the four-factor full factorial design.

Source	Degrees of Freedom	Sum of Squares	Mean Squares
Factor *A*	$\left(l_{1}-1\right)$	$\sum_{i}l_{2}l_{3}l_{4}{\left({\bar{y}}_{i...}-{\bar{y}}_{....}\right)}^{2}$	$SS_{A}/df_{A}$
Factor *B*	$\left(l_{2}-1\right)$	$\sum_{j}l_{1}l_{3}l_{4}{\left({\bar{y}}_{.j..}-{\bar{y}}_{....}\right)}^{2}$	$SS_{B}/df_{B}$
Factor *C*	$\left(l_{3}-1\right)$	$\sum_{k}l_{1}l_{2}l_{4}{\left({\bar{y}}_{..k.}-{\bar{y}}_{....}\right)}^{2}$	$SS_{C}/df_{C}$
Factor *D*	$\left(l_{4}-1\right)$	$\sum_{l}l_{1}l_{2}l_{3}{\left({\bar{y}}_{...l}-{\bar{y}}_{....}\right)}^{2}$	$SS_{D}/df_{D}$
Factor AB	$\left(l_{1}-1\right)\left(l_{2}-1\right)$	$\sum_{ij}l_{3}l_{4}{\left({\bar{y}}_{ij..}-{\bar{y}}_{i...}-{\bar{y}}_{.j..}-{\bar{y}}_{....}\right)}^{2}$	$SS_{AB}/df_{AB}$
Factor AC	$\left(l_{1}-1\right)\left(l_{3}-1\right)$	$\sum_{ik}l_{2}l_{4}{\left({\bar{y}}_{i.k.}-{\bar{y}}_{i...}-{\bar{y}}_{..k.}-{\bar{y}}_{....}\right)}^{2}$	$SS_{AC}/df_{AC}$
Factor AD	$\left(l_{1}-1\right)\left(l_{4}-1\right)$	$\sum_{il}l_{2}l_{3}{\left({\bar{y}}_{i..l}-{\bar{y}}_{i...}-{\bar{y}}_{...l}-{\bar{y}}_{....}\right)}^{2}$	$SS_{AD}/df_{AD}$
Factor BC	$\left(l_{2}-1\right)\left(l_{3}-1\right)$	$\sum_{jk}l_{1}l_{4}{\left({\bar{y}}_{.jk.}-{\bar{y}}_{.j..}-{\bar{y}}_{..k.}-{\bar{y}}_{....}\right)}^{2}$	$SS_{BC}/df_{BC}$
Factor BD	$\left(l_{2}-1\right)\left(l_{4}-1\right)$	$\sum_{jl}l_{1}l_{3}{\left({\bar{y}}_{.j.l}-{\bar{y}}_{.j..}-{\bar{y}}_{...l}-{\bar{y}}_{....}\right)}^{2}$	$SS_{BD}/df_{BD}$
Factor CD	$\left(l_{3}-1\right)\left(l_{4}-1\right)$	$\sum_{kl}l_{1}l_{2}{\left({\bar{y}}_{.k.l}-{\bar{y}}_{..k.}-{\bar{y}}_{...l}-{\bar{y}}_{....}\right)}^{2}$	$SS_{CD}/df_{CD}$
Factor ABC	$\left(l_{1}-1\right)\left(l_{2}-1\right)\left(l_{3}-1\right)$	$\sum_{ijk}l_{4}{\left({\bar{y}}_{ijk.}-{\bar{y}}_{i...}-{\bar{y}}_{.j..}-{\bar{y}}_{..k.}-{\bar{y}}_{....}\right)}^{2}$	$SS_{ABC}/df_{ABC}$
Factor ABD	$\left(l_{1}-1\right)\left(l_{2}-1\right)\left(l_{4}-1\right)$	$\sum_{ijl}l_{3}{\left({\bar{y}}_{ij.l}-{\bar{y}}_{i...}-{\bar{y}}_{.j..}-{\bar{y}}_{...l}-{\bar{y}}_{....}\right)}^{2}$	$SS_{ABD}/df_{ABD}$
Factor ACD	$\left(l_{1}-1\right)\left(l_{3}-1\right)\left(l_{4}-1\right)$	$\sum_{ikl}l_{2}{\left({\bar{y}}_{i.kl}-{\bar{y}}_{i...}-{\bar{y}}_{..k.}-{\bar{y}}_{...l}-{\bar{y}}_{....}\right)}^{2}$	$SS_{ACD}/df_{ACD}$
Factor BCD	$\left(l_{2}-1\right)\left(l_{3}-1\right)\left(l_{4}-1\right)$	$\sum_{jkl}l_{1}{\left({\bar{y}}_{.jkl}-{\bar{y}}_{.j..}-{\bar{y}}_{..k.}-{\bar{y}}_{...l}-{\bar{y}}_{....}\right)}^{2}$	$SS_{BCD}/df_{BCD}$
Residual	$l_{1}l_{2}l_{3}l_{4}\left(n-1\right)$	Difference	$SS_{E}/df_{E}$
Corrected Total	$l_{1}l_{2}l_{3}l_{4}-1$	$\sum_{ijkl}{\left(y_{ijkl}-{\bar{y}}_{....}\right)}^{2}$	$SS_{CT}/df_{CT}$

**Table 4:** Modulus of elasticity Rresults for the four-factor ANOVA.

Source	d.f.	Sum Sq.	Mean Sq.	F	*p*	Conclusion *
A	2	3.3016 x 10${}^{7}$	1.6508 × 10${}^{7}$	576.08	1.24 × 10${}^{-15}$	S
B	2	3.4356 × 10${}^{7}$	1.7178 × 10${}^{7}$	599.46	9.05 × 10${}^{-16}$	S
C	2	1.4900 × 10${}^{6}$	7.4502 × 10${}^{5}$	26.00	9.40 × 10${}^{-6}$	S
D	2	9.0965 × 10${}^{7}$	4.5482 × 10${}^{7}$	1587.20	4.00 × 10${}^{-19}$	S
AB	4	2.6466 × 10${}^{6}$	6.6165 × 10${}^{5}$	23.09	1.77 × 10${}^{-6}$	S
AC	4	3.2190 × 10${}^{5}$	8.0476 × 10${}^{4}$	2.81	0.06	NS
AD	4	7.4523 × 10${}^{6}$	1.8631 × 10${}^{6}$	65.02	1.09 × 10${}^{-9}$	S
BC	4	1.3519 × 10${}^{5}$	3.3798 × 10${}^{4}$	1.18	0.36	NS
BD	4	5.8460 × 10${}^{6}$	1.4615 × 10${}^{6}$	51.00	6.57 × 10${}^{-9}$	S
CD	4	7.5876 × 10${}^{4}$	1.8969 × 10${}^{4}$	0.66	0.63	NS
ABC	8	1.2613 × 10${}^{5}$	1.5766 × 10${}^{4}$	0.55	0.80	NS
ABD	8	3.9104 × 10${}^{5}$	4.8879 × 10${}^{4}$	1.71	0.17	NS
ACD	8	1.7275 × 10${}^{5}$	2.1593 × 10${}^{4}$	0.75	0.65	NS
BCD	8	3.6980 × 10${}^{5}$	4.6225 × 10${}^{4}$	1.61	0.19	NS
Residuals	16	4.5849 × 10${}^{5}$	2.8656 × 10${}^{4}$			
Total	80	1.7782 × 10${}^{8}$				

* Conclusion column represents statistical significance (S) or non-significance (NS) of the factor or interaction based on α-level of 0.01.

**Table 5:** Yield Strength Results for the Four-Factor ANOVA.

Source	d.f.	Sum Sq.	Mean Sq.	F	*p*	Conclusion *
A	2	1.0610 × 10${}^{4}$	5305.12	525.97	2.54 × 10${}^{-15}$	S
B	2	2.3371 × 10${}^{4}$	1.1685 × 10${}^{4}$	1168.51	4.89 × 10${}^{-18}$	S
C	2	20.1641	10.0821	1.00	0.39	NS
D	2	4.3981 × 10${}^{4}$	2.1990 × 10${}^{4}$	2180.20	3.19 × 10${}^{-20}$	S
AB	4	2330.34	582.5722	57.76	2.63 × 10${}^{-9}$	S
AC	4	90.5071	22.6268	2.24	0.11	NS
AD	4	4408.57	110.22	109.27	2.11 × 10${}^{-11}$	S
BC	4	9.6451	2.4113	0.24	0.91	NS
BD	4	7051.41	1762.88	174.78	5.54 × 10${}^{-13}$	S
CD	4	21.1121	5.2780	0.52	0.72	NS
ABC	8	22.9918	2.8740	0.28	0.96	NS
ABD	8	910.6680	113.8335	11.29	2.83 × 10${}^{-5}$	S
ACD	8	78.8200	9.8525	0.98	0.49	NS
BCD	8	96.6111	12.0764	1.20	0.36	NS
Residuals	16	161.3825	10.0864			
Total	80	9.3164 × 10${}^{4}$				

* Conclusion column represents statistical significance (S) or non-significance (NS) of the factor or interaction based on α-level of 0.01.

**Table 6:** Plateau stress results for the four-factor ANOVA.

Source	d.f.	Sum Sq.	Mean Sq.	F	*p*	Conclusion *
A	2	1.4838 × 10${}^{5}$	7.4190 × 10${}^{4}$	5545.52	1.85 × 10${}^{-23}$	S
B	2	1.1620 × 10${}^{5}$	5.8100 × 10${}^{4}$	4342.80	1.31 × 10${}^{-22}$	S
C	2	280.4803	140.2402	10.48	0.01	S
D	2	1.8137 × 10${}^{5}$	9.0687 × 10${}^{4}$	6778.53	3.73 × 10${}^{-24}$	S
AB	4	3.4732 × 10${}^{4}$	8683.10	649.03	1.77 × 10${}^{-17}$	S
AC	4	45.0679	11.2670	0.84	0.52	NS
AD	4	5.4646 × 10${}^{4}$	1.3661 × 10${}^{4}$	1021.11	4.82 × 10${}^{-19}$	S
BC	4	30.1069	7.5267	0.56	0.69	NS
BD	4	4.2436 × 10${}^{4}$	1.0609 × 10${}^{4}$	792.99	3.61 × 10${}^{-18}$	S
CD	4	97.5850	24.3963	1.82	0.17	NS
ABC	8	47.8212	5.9776	0.45	0.88	NS
ABD	8	1.3934 × 10${}^{4}$	1741.83	130.19	4.35 × 10${}^{-13}$	S
ACD	8	101.6527	12.7066	0.95	0.50	NS
BCD	8	160.4661	20.0583	1.50	0.23	NS
Residuals	16	214.0562	13.3785			
Total	80	5.9268 × 10${}^{5}$				

* Conclusion column represents statistical significance (S) or non-significance (NS) of the factor or interaction based on α-level of 0.01.

**Table 7:** Toughness results for the four-factor ANOVA.

Source	d.f.	Sum Sq.	Mean Sq.	F	*p*	Conclusion *
A	2	9.2034 x 10${}^{4}$	4.6017 x 10${}^{4}$	86.93	2.54 x 10${}^{-9}$	S
B	2	8.6882 × 10${}^{4}$	4.3441 × 10${}^{4}$	82.07	3.87 × 10${}^{-9}$	S
C	2	3248.39	1624.21	3.07	0.07	NS
D	2	1.0513 × 10${}^{5}$	5.2565 × 10${}^{4}$	99.30	9.55 × 10${}^{-10}$	S
AB	4	2.9799 × 10${}^{4}$	7449.71	14.07	4.16 × 10${}^{-5}$	S
AC	4	8395.56	2098.87	3.97	0.02	NS
AD	4	2.7039 × 10${}^{4}$	6759.82	12.77	7.43 × 10${}^{-5}$	S
BC	4	1852.48	463.1330	0.87	0.50	NS
BD	4	3.3487 × 10${}^{4}$	8371.67	15.82	2.04 × 10${}^{-5}$	S
CD	4	7869.07	1967.33	3.72	0.03	NS
ABC	8	3439.51	429.9413	0.81	0.60	NS
ABD	8	1.1765 × 10${}^{4}$	1470.68	2.78	0.04	NS
ACD	8	1.8175 × 10${}^{4}$	2271.80	4.29	0.01	NS
BCD	8	3944.19	493.0227	0.93	0.52	NS
Residuals	16	8469.37	529.3384			
Total	80	4.4153 × 10${}^{5}$				

* Conclusion column represents statistical significance (S) or non-significance (NS) of the factor or interaction based on α-level of 0.01.

**Table 8:** Modulus of elasticity results for the reduced model four-factor ANOVA.

Source	d.f.	Sum Sq.	Mean Sq.	F	*p*	Conclusion *
A	2	3.30 x 10${}^{7}$	1.65 x 10${}^{7}$	482.89	1.03 x 10${}^{-37}$	S
B	2	3.44 × 10${}^{7}$	1.72 × 10${}^{7}$	502.49	3.35 × 10${}^{-38}$	S
C	2	1.49 × 10${}^{6}$	7.45 × 10${}^{5}$	21.79	7.68 × 10${}^{-8}$	S
D	2	9.10 × 10${}^{7}$	4.55 × 10${}^{7}$	1330.44	2.01 × 10${}^{-50}$	S
AB	4	2.65 × 10${}^{6}$	6.62 × 10${}^{5}$	19.35	2.86 × 10${}^{-10}$	S
AD	4	7.45 × 10${}^{6}$	1.86 × 10${}^{6}$	54.50	2.59 × 10${}^{-19}$	S
BD	4	5.85 × 10${}^{6}$	1.46 × 10${}^{6}$	42.75	6.33 × 10${}^{-17}$	S
Residuals	60	2.05 × 10${}^{6}$	3.42 × 10${}^{4}$			
Total	80	1.78 × 10${}^{8}$				

* Conclusion column represents statistical significance (S) or non-significance (NS) of the factor or interaction based on α-level of 0.01.

**Table 9:** Yield strength results for the reduced model four-factor ANOVA.

Source	d.f.	Sum Sq.	Mean Sq.	F	*p*	Conclusion *
A	2	1.06 × 10${}^{4}$	5310.28	228.71	8.49 × 10${}^{-29}$	S
B	2	2.34 × 10${}^{4}$	1.17 × 10${}^{4}$	503.77	3.11 × 10${}^{-38}$	S
C	2	20.16	10.08	0.43	0.65	NS
D	2	4.40 × 10${}^{4}$	2.20 × 10${}^{4}$	948.04	4.01 × 10${}^{-46}$	S
AB	4	2334.74	582.57	25.12	3.02 × 10${}^{-12}$	S
AD	4	4410.59	110.47	47.52	6.02 × 10${}^{-18}$	S
BD	4	7048.92	1757.28	76.00	8.46 × 10${}^{-23}$	S
Residuals	60	1391.27	23.20			
Total	80	9.32 × 10${}^{4}$				

* Conclusion column represents statistical significance (S) or non-significance (NS) of the factor or interaction based on α-level of 0.01.

**Table 10:** Plateau stress results for the reduced model four-factor ANOVA.

Source	d.f.	Sum Sq.	Mean Sq.	F	*p*	Conclusion *
A	2	1.48 × 10${}^{5}$	7.42 × 10${}^{4}$	304.24	3.91 × 10${}^{-32}$	S
B	2	1.16 × 10${}^{5}$	5.81 × 10${}^{4}$	238.26	2.87 × 10${}^{-29}$	S
C	2	280.48	140.24	0.58	0.57	NS
D	2	1.81 × 10${}^{5}$	9.07 × 10${}^{4}$	371.89	1.55 × 10${}^{-34}$	S
AB	4	3.47 × 10${}^{4}$	8678.23	35.61	3.17 × 10${}^{-15}$	S
AD	4	5.46 × 10${}^{4}$	1.37 × 10${}^{4}$	56.02	1.36 × 10${}^{-19}$	S
BD	4	4.24 × 10${}^{4}$	1.06 × 10${}^{4}$	43.51	4.31 × 10${}^{-17}$	S
Residuals	60	1.46 × 10${}^{4}$	243.85			
Total	80	5.93 × 10${}^{5}$				

* Conclusion column represents statistical significance (S) or non-significance (NS) of the factor or interaction based on α-level of 0.01.

**Table 11:** Toughness results for the reduced model four-factor ANOVA.

Source	d.f.	Sum Sq.	Mean Sq.	F	*p*	Conclusion *
A	2	9.20 × 10${}^{4}$	4.60 × 10${}^{4}$	43.20	2.39 × 10${}^{-12}$	S
B	2	8.69 × 10${}^{4}$	4.34 × 10${}^{4}$	40.78	6.54 × 10${}^{-12}$	S
C	2	3252.83	1618.70	1.52	0.23	NS
D	2	1.05 × 10${}^{5}$	5.26 × 10${}^{4}$	49.35	2.13 × 10${}^{-13}$	S
AB	4	2.98 × 10${}^{4}$	7451.64	6.99	1.09 × 10${}^{-4}$	S
AD	4	2.70 × 10${}^{4}$	6759.02	6.35	2.51 × 10${}^{-4}$	S
BD	4	3.35 × 10${}^{4}$	8371.48	7.86	3.67 × 10${}^{-5}$	S
Residuals	60	6.39 × 10${}^{4}$	1070.39			
Total	80	4.42 × 10${}^{5}$				

* Conclusion column represents statistical significance (S) or non-significance (NS) of the factor or interaction based on α-level of 0.01.

**Table 12:** Least squares main factor contrasts based on reduced interaction model.

Factor	Level	Estimate			
		Elastic Modulus (MPa)	Yield Strength (MPa)	Plateau Stress (MPa)	Toughness (MJ/m^3^)
Lattice Design	Diamond	−242.04	−8.28	−28.61	−24.52
	I-WP	874.33	16.18	60.50	47.66
	Primitive	−632.29	−7.91	−31.89	−23.14
Cell Size (mm)	4	864.00	22.93	51.59	45.04
	6	−155.68	−5.26	−13.32	−13.15
	8	−708.68	−17.67	−38.27	−31.88
Cell Density	3	−151.35	−0.68	−2.03	4.22
	4	−26.37	0.18	−0.44	4.73
	6	177.72	0.50	2.47	−8.95
Surface Thickness (mm)	0.2	−1282.52	−26.54	−52.58	−41.07
	0.4	−30.22	−3.65	−9.56	−5.57
	0.6	1312.75	30.19	62.14	46.64
Response Means		2489.38	40.70	75.41	59.93

**Table 13:** 95% confidence intervals based on reduced interaction model.

Mechanical Property	Interval
Elastic Modulus	54.65
Yield Strength	1.42
Plateau Stress	4.62
Toughness	9.65

## Data Availability

Data will be made available upon reasonable request.

## Abbreviations

The following abbreviations are used in this manuscript:

ANOVA	Analysis of Variance
ASTM	American Society for Testing and Materials
CRD	Completely Randomized Design
DOE	Design of Experiments
IN718	Inconel 718
SLM	Selective Laser Melting
TPMS	Triply Periodic Minimal Surfaces
